# Can cochlear implantation prevent cognitive decline in the long-term follow-up?

**DOI:** 10.3389/fneur.2022.1009087

**Published:** 2022-10-20

**Authors:** Christiane Völter, Lisa Götze, Stefan Thomas Kamin, Imme Haubitz, Stefan Dazert, Jan Peter Thomas

**Affiliations:** ^1^Department of Otorhinolaryngology, Head and Neck Surgery, Catholic Hospital Bochum, Ruhr-University Bochum, Bochum, Germany; ^2^Department of Psychology, Institute of Psychogerontology, Friedrich-Alexander University Erlangen-Nürnberg, Nuremberg, Germany; ^3^Department of Otorhinolaryngology, Head and Neck Surgery, St.-Johannes-Hospital, Dortmund, Germany

**Keywords:** dementia, hearing loss, prevention, cochlear implant, auditory rehabilitation

## Abstract

Cognitive function and hearing are known to both decline in older adults. As hearing loss is proposed to be one modifiable risk factor for dementia, the impact of auditory rehabilitation on cognitive decline has been gaining increasing attention. Despite a large number of studies, long-term data are still rare. In a large prospective longitudinal monocentric study, 50 adults (aged ≥ 50 years) with severe postlingual bilateral hearing loss received a cochlear implant (CI). They underwent comprehensive neurocognitive testing prior to implantation (T1), at 12 months (T2) and up to 65 months (T3) after implantation. Various cognitive subdomains such as attention, inhibition, working memory, verbal fluency, mental flexibility and (delayed) recall were assessed by the computer-based non-auditory test battery ALAcog^©^. The observed trajectories of two exemplary cognitive subdomains (delayed recall and working memory) were then fitted over time using multilevel growth models to adjust for sociodemographic covariates and compared with 5-year longitudinal data from a sample of older adults from the representative Survey of Health, Aging and Retirement in Europe (SHARE) study. Postoperatively, auditory functions improved from 6.98% (SD 12.83) to 57.29% (SD 20.18) in monosyllabic speech understanding. Cognitive functions significantly increased from T1 to T3 in attention (*p* = 0.001), delayed recall (*p* = 0.001), working memory (OSPAN; *p* = 0.001), verbal fluency (*p* = 0.004), and inhibition (*p* = 0.002). A closer look at follow-up revealed that cognitive improvement could be detected between T1 and T2 and thereafter remained stable in all subtests (*p* ≥ 0.06). Additional longitudinal analysis confirmed these findings in a rigorous multilevel approach in two exemplary cognitive subdomains. In contrast to the SHARE data, there was no evidence for age-differential associations over time in CI recipients. This suggests that older adults benefit equally from cochlear implantation. CI users with worse preoperative cognitive skills experienced the most benefit (*p* < 0.0001). Auditory rehabilitation by cochlear implantation has a stimulating effect on cognitive functions beyond an improvement in speech understanding and an increased well-being. Large multicenter studies using standardized protocols have to be undertaken in the future to find out whether hearing restoration might help to prevent cognitive decline.

## Introduction

With more people living longer, the issue of healthy aging is of increasing importance (1). In addition to preserving a good physical constitution, maintaining cognitive function is quite important (2). According to the World Health Organization, more than 55 million people currently suffer from dementia worldwide and this number is predicted to rise to 78 million by 2030, and to 139 million by 2050 (3). As no causal treatment exists to reverse cognitive decline, efforts must focus on prevention (4). Recently, 12 risk factors in midlife were identified that account for 40% of dementia risk. Hearing loss is one of them (5). Livingston et al. estimated that appropriate treatment of hearing loss can reduce the prevalence of dementia by 8% (5). Therefore, the question arises: can auditory rehabilitation *via* hearing devices in middle age delay or even reverse cognitive decline (6, 7).

During the last decade studies have analyzed the benefit of hearing aids on cognitive performance; however, data were heterogeneous (8–11). In severe hearing loss cochlear implants (CI) are the option of choice (12–14). However, only 8.5% of people who would benefit from a CI actually receive one (15). This is alarming, as people with severe hearing loss are at a 4.94% higher risk of developing dementia than people with mild hearing loss (1.89%) (16).

Thus, people with a severe to profound hearing loss are of special interest in terms of preventing cognitive decline with age. To this end, a number of studies have been performed recently on cognitive changes after cochlear implantation (17–22). Mosnier et al. were among the first who evaluated, in a multicenter study, the cognitive function on 91 CI candidates, classified into normal and abnormal based on normative data from six different cognitive tests (23). 20% of the subjects aged 65–85 years had an abnormal score on at least three out of six subtests before CI provision; this decreased to only 5% after cochlear implantation. In general, cognitive functions significantly improved at six or at 12 months of CI use (22, 24). This is in line with data reporting on the improvement in speech perception (25). However, the effect size was smaller and the results were different for each subtype of cognitive function (26).

Despite these promising findings, data are not yet conclusive due to the large heterogeneity across the studies, as the test material and the study protocols used were mainly based on in-house standards and thus hard to compare (27). Whereas some studies used a single-center design (18, 20, 26, 28, 29) other authors collected data in multi-center settings (30–32) in different countries or even different languages and inconsistency in data sets due to language or cultural background cannot be ruled out (33). In addition to differences in subjects' ages, a huge number of different cognitive assessments were applied (34). Some studies used test batteries covering only a few cognitive domains or screening tests which might have overlooked slight cognitive changes (17, 24, 29, 35). Others applied auditory-based test material which was not suitable for people with severe hearing loss (23, 32). Despite authors' claim that audibility was ensured, misunderstanding cannot fully be excluded because verbally based cognitive tests may be influenced by auditory deprivation and can cause false positive results in up to 16% of tests (36–38).

Another challenge is that follow-up intervals were quite short. Most researchers published data on a follow-up interval of ≤ 12 months after cochlear implantation (24, 29). So far, only two studies have analyzed data on a much longer follow-up (28, 32). Cosetti et al. published data in seven female CI recipients after a mean follow-up of 3.7 years, ranging between 2 and 4.1 years (28). The longest follow-up measure was provided by Mosnier et al. in 70 CI recipients (32). Building up on their initial sample (23), data on preoperative and 1-year performance as well as a third assessment which took place 5–8.5 years after implantation were reported (32).

Most studies lack a suitable control group due to ethical reasons, as it would be unethical to deny hearing devices to people with severe hearing loss (6, 18, 27, 39). In the few studies that did include controls, the effect of age and education was not controlled for (19) or the number of control subjects included was quite small considering the huge variability in cognitive performance in older age (30, 31, 40–43). Furthermore, in most studies mean cognitive changes were evaluated for the whole study group, but not on an individual level. Only a few have analyzed the performance of individuals themselves (23, 28, 44).

In other words, we know that CI users perform better in some neurocognitive domains shortly after cochlear implantation, but we do not know if cochlear implantation can reverse the general cognitive decline in individual users in the long-term follow-up (18). This is highly relevant because it takes a couple of years for mild cognitive impairment to develop into dementia (45) and it is hard to differentiate healthy physiological aging from a pathological process (41) because there is a huge variability in cognitive performance in age (40) and cognitive decline is (a) influenced by major environmental, psychosocial and biological factors and (b) not linear.

From the perspective of cognitive aging research, benefits of CI use on cognition are expected. Robust evidence exists proving the plasticity of the aging brain (46, 47). One potential pathway to explain such plasticity is the cognitive stimulation provided by social and physical environments (48). Studies have indicated positive effects of a socially and physically active lifestyle on cognition among healthy older adults (49–51) and even subjects with dementia (52). Accordingly, reversing deficits in hearing after cochlear implantation may have a direct impact on cognition through the experience of richer and cognitively more stimulating environments (44).

Therefore, the aims of the study were firstly, to assess cognitive function before and after long-term CI use in a prospective single-center approach in a large sample of CI users. Focus will be put on the users as a whole and also on users as individuals. And secondly, to compare average trajectories in cognitive abilities in CI users with a sample of older adults from the representative SHARE (Survey of Health, Ageing and Retirement in Europe) study as a control group. This was done to approximate the effect of CI use on specific cognitive domains in the absence of a control condition.

## Materials and methods

This study was approved by the ethics institution of the Ruhr-University Bochum (No. 16-5727-BR). The study meets the guidelines set forth in the Declaration of Helsinki. All participants gave their written consent.

We describe the methods separately for primary and secondary data. The primary data analyses are based on the analytical state-of-art commonly used in biomedical research by comparing differences and exploring intraindividual clinical trajectories in cognitive measures over time. The secondary analyses contribute to this approach in two ways: firstly, the SHARE data allows us to explore effects of cochlear implantation on exemplary cognitive domains in relation to observed changes in a specific population; secondly, the statistical approach used to compare both datasets is more rigorous, adding further robustness regarding the primary data. For example, multilevel models use all data, account for correlations of repeated measures, and are robust against differences in length of follow-up (53). Moreover, this approach accounts for variability at the individual subject level, which may otherwise introduce bias when estimating changes in cognition over time.

### Primary data

#### Participants/study samples

Since 2016 CI candidates aged ≥50 years presented at the comprehensive hearing center, Ruhr-University Bochum, were screened for study participation according to pre-defined inclusion/exclusion criteria (21, 26). Seventy one subjects performed cognitive assessment prior (T1) to as well as 12 months post cochlear implantation (T2). 50 CI recipients who had been implanted at least 42 months before (mean follow-up of 4.5 years, SD 0.5) were re-assessed at T3; 21 had to be excluded due to: critical health conditions (*n* = 5), death (*n* = 2), unwillingness to participate further (*n* = 4), relocation (*n* = 4), or loss to follow-up (*n* = 6). Only the 50 subjects who underwent testing at T1 as well as at T2 and at T3 were included in the data analysis. Educational level was assessed by the number of educational years and grouped according to the International Standard Classification of Education 2011 (54). Level 1 represents primary education; level 2 lower secondary education; level 3 upper secondary education; level 4 post-secondary, non-tertiary education; level 5 first stage of tertiary education; and level 6 second stage of tertiary education. Participants' demographic data are summarized in Table 1.

**Table 1:** Sociodemographic data on the cochlear implant recipients and the SHARE sample.

	**Cochlear implant**		**SHARE sample**	
	**recipients**		
	**M (SD) or %**	**Range**	**M (SD) or %**	**Range**
Age	63.98 (9.13)	50–81	64.74 (5.86)	51–81
Male	38%	0–1	47%	0–1
Education^a^	2.84 (0.77)	2–4	2.83 (0.60)	2–4
Memory^b^	0.00 (1.00)	−1.55 to 1.16	0.00 (1.00)	−2.28 to 2.86
Working	0.00 (1.00)	−1.40 to 1.40	0.00 (1.00)	−4.05 to 0.51
memory^c^				

a ISCED-2011 coded educational level (0 = lowest to 6 = highest).

b Standardized scores of delayed recall across all measurements.

c Standardized scores of Serial 7s (SHARE) and OSPAN (CI recipients) across all measurements.

#### Audiometric assessment

Preoperatively, pure-tone thresholds were measured for each ear at 0.25–8 kHz in a soundproof booth (DIN EN ISO 8253). Speech understanding in quiet was assessed *via* the German language Freiburg monosyllabic speech test at 65 dB sound pressure level (SPL) at three intervals: preoperatively and 12 months and up to 65 months after implantation. Postoperatively, tests were performed in CI-only testing condition. All testing was conducted by an experienced audiologist.

#### Neurocognitive assessment

Subjects underwent a cognitive evaluation preoperatively (T1), 12 (T2) and up to 65 months (T3) after cochlear implantation with a mean T3 follow-up of 4.5 (SD 0.5) years described as 5-year data. In a few cognitive subtests data were not available for all subjects at each assessment. Therefore, sample size varied in the different subdomains. Neurocognitive testing was done by the computer-based neurocognitive assessment tool ALAcog, which consists of nine subtests covering the following cognitive domains, as described in detail by Falkenstein et al. and by Völter et al. (55, 56): in the M3 test, which assesses attention, a target letter and some distractors are presented, and the target has to be clicked as fast as possible. In the recall and the delayed recall task, 10 words are shown which have to be memorized immediately and after 30 min. For working memory, (1) the 2-back task was used, where a reaction is required in case the letter shown is identical to the second last, (2) further the Operation Span (OSPAN) task. In this dual task, letters have to be memorized, while equations have to be performed. The Flanker test measures the ability to suppress and to inhibit stimuli. The participant is asked to respond to a target flanked by arrow pointers above and underneath pointing in the same (compatible Flanker) or in different directions (incompatible Flanker). Two Trail Making Test (TMT) tasks were also included: the TMT A, which measures simple processing speed, and the TMT B, which assesses executive function. In both TMTs, participants have to sort randomly shown items as quickly as possible, in TMT A numbers from 1 to 26 and in TMT B numbers from 1 to 13 and letters from A to M. In the verbal fluency task, as many animals as possible starting with a particular letter have to be named within 90 s.

A total score, the inverse efficiency (IE), was calculated based on the time needed and the number of correct answers given. A lower IE score indicated a better performance. Practice effects were minimized by different test versions.

#### Questionnaires

The **Nijmegen Cochlear Implant Questionnaire (NCIQ)** was used to evaluate the health-related quality of life (HRQoL) (57). A total score was calculated from three domains, (1) *physical domain*: (a) basic sound perception, (b) advanced sound perception, and (c) speech production; (2) *psychological domain*: (a) self-esteem; (3) *social domain*: (a) activity limitations and (b) social interactions. A higher score indicates better HRQoL. The **Cognitive Reserve Index Questionnaire (CRIq)** was used to assess cognitive reserve (CR) throughout lifetime including several psychosocial and environmental factors: (1) education, (2) leisure time, and (3) working activity, and the demographic data. A total score is calculated by combining the three subdomains adjusted for age. A score < 70 points represents a low CR, 70–84 a medium-low CR, 85–114 a medium CR, 115–130 a medium-high CR, and >130 a high CR (58). Depressive symptoms were questioned by the **Geriatric Depression Scale 15 (GDS-15)** (59). A score of 0–5 points indicates no depressive symptoms, 6–10 points indicates slight to moderate symptoms, and ≥11 points indicates severe depression.

#### Statistical analysis

Statistical analysis was done by Medas (Grund, Margetshochheim, Germany). First, data were tested for distribution. In case of non-parametric data (all cognitive subtests, except the recall task), the median and the 68% confidence interval, and in case of parametric data (NCIQ, CRIq, GDS-15, duration of hearing aid use, duration of deafness, and speech perception), mean and standard deviation were reported. In order to provide consistency in cognitive data, also the median of the recall was reported. For all data, rank correlation between two variables were calculated by using Kendall's τ. To compare pre- and postoperative results, the Wilcoxon-test and the Mann-Whitney-*U* test were used to analyze the different groups. If a participant was not able to finish the TMT test within 3 min, the rule of proportion was applied.

Multiregression analysis based on educational background, sex, and cognitive baseline score was done to discover which variable is the most predictive regarding cognitive performance at T3. Cohen's d was used for the calculation of effect sizes (*d* = 0.2– 0.4 is a small, *d* = 0.5– 0.7 a medium, and *d* ≥ 0.8 a large effect size) for parametric data and after transformation for non-parametric data. To analyze the individual performance first data transformation for each subtest (M3, delayed recall, Flanker and OSPAN) was calculated for a parametric distribution. Later on, the standard error of the mean (SEM) was calculated. A score which was below the mean ± of the SEM, was considered as an improvement. A score that was higher indicated a poorer performance, a score within the range of the mean ± of the SEM was considered as a stable performance. Statistical significance was set to *p* < 0.05. To correct for multiple comparisons, Bonferroni correction was applied with *p* < 0.005.

### Secondary data

#### Participants/study samples

Secondary data analyses were done based on the Survey of Health, Ageing and Retirement in Europe (SHARE), an ongoing cross-national representative panel study of ≥50 years old adults which addresses various key areas of individual and social aging including health variables, socio-economic information, social networks, physical measures, biomarkers, and psychological variables. Episodic memory (delayed recall) and working memory (Serial 7s task) are two cognitive key domains of the neurobiologically based cognitive mechanics that typically show age-related declines in later life (60, 61). They were assessed in the SHARE study and in the primary data selected for detailed analysis. A detailed summary of SHARE sampling procedures and study design is described in Börsch-Supan et al. (62).

For the current study, we used three waves with an observational period of 5 years (T1: 2015; T2: 2017, and T3: 2020). We followed a two-stage procedure to ensure comparability with the primary data. Firstly, we included SHARE participants who: (a) were at least 50 years and no more than 81 years old at time of first assessment and (b) had an International Standard Classification of Education (ISCED) range between level 2 (lower-secondary education) and level 4 (post-secondary education). We excluded SHARE participants who (a) reported diagnoses of cognitive impairments or other neurological diseases such as Alzheimer's or Parkinson's disease, and (b) suffered from depression as per a scale score of 4 or higher on the EURO-D scale (63). This resulted in a sample of 2,709 participants who provided full information on the data.

Secondly, we used this sample and applied sampling weights for chronological age to draw a random sample of 1,000 participants to match the age distribution of the primary data. The resulting sample provided a mean age of 64.74 (SD 5.86) years and a mean ISCED level of 2.83 (SD.60). Two-sample *t*-tests confirmed that there was no statistical difference between SHARE participants and CI recipients. Table 1 provides a description of the sample.

#### Neurocognitive assessment

SHARE includes various cognitive measures at each wave. We selected two measures that assessed central cognitive domains that were also measured in the participants with a CI, namely (delayed) recall of a 10-word list to measure short and long-term memory and the Serial 7s task to evaluate working memory capacity and attention. For the delayed recall test, participants listened to a list of 10 words and were asked to recall the list immediately (first trial) and once after a delay time of ~10 min. For the Serial 7s task, participants had to count backwards from 100 by 7s, stopping after the fifth answer. We standardized both test scores with higher values reflecting better cognitive performance. For a detailed description of the survey measures see Dewey and Prince (64).

#### Covariates

These included chronological age in years, sex (0 = female; 1 = male), and highest educational level by ISCED-2011. The SHARE data did not include objective audiometric assessments, which may have introduced bias with regard to the influence of hearing impairment on cognitive trajectories. We performed supplementary analyses and included a covariate of subjective evaluation of hearing to account for this issue.

#### Statistical analysis

Statistical analysis was conducted with R 4.2.1. (65). In order to compare the SHARE and the cochlear implanted participants, cognitive tests were standardized on their means and SDs for each sample and at each measurement. The raw scores of CI participants were transformed into a reverse-coded 5-point scale prior to standardization. This was necessary to harmonize the interpretation of the standardized scores between the samples (i.e., higher values indicate better cognitive performance). We estimated fixed-effects multilevel growth models for each study and outcome using the nlme package (66). The models included measurement occasions (level 1) nested within participants (level 2) to assess the effect of time on change in the cognitive outcomes across the samples. We used this approach because it easily handles unbalanced data with uneven time points (67), which is the case for the CI group. Time X age interactions were also included to test whether change over time depended on the age of the participants. The interactions were illustrated by plotting time slopes at two different mean values of chronological age based on median splits in the respective samples. These values reflect two age-categories that were defined as “young-old adults” (SHARE sample: 51–63 years; cochlear sample: 50–66 years) and “old-old adults” (SHARE sample: 64 to 81 years; cochlear sample: 67–81 years). Please note that these categories were empirically derived from the respective samples and only used for analytical purposes to illustrate the overall direction of the interaction effects. This method is recommended by Preacher and colleagues to facilitate the interpretation of interaction terms and is widely used in empirical research (68). All models were controlled for chronological age, sex, and educational status as time-independent predictors at level 2. Chronological age and educational status were centered around the mean for each study to make the intercepts interpretable. The time variable was recorded (i.e., 0 = T1; 1 = T2; 2 = T3) to ensure that intercepts reflect predicted values of cognitive measures at the first measurement. Therefore, change in the slope factor was interpreted as the average change for each additional measurement within the respective samples.

## Results

### Audiometric data

Mean 4-PTA of the better ear was 88.15 (SD 18.95) dB and for the poorer ear 98.2 (SD 15.55) dB at T1. On average, subjects suffered from a severe to profound hearing loss for 21.43 (SD 13.92) years prior to implantation. Preoperatively, subjects' mean unaided monosyllabic speech perception was 5.12% (SD 10.05) at 65 dB for the ear to be implanted. Speech perception in quiet at 65 dB significantly improved from 6.98% (SD 12.83) at T1 (with hearing aids) to 57.29% (SD 20.18) at T2 (*p* < 0.0001) and remained stable at 54.39% (SD 20.04) at T3. No further benefit was found between T2 and T3 at 65 dB (*p* = 0.46). Regarding gender, men had significantly better scores at T1 (men 13.0 (SD 17.2); women 3.33 (SD 6.86); *p* = 0.03) and T3 [men 66.75 (SD 11.62); women 45.86 (SD 20.31); *p* = 0.0001]. Improvement in speech perception between T1 and T3 was greater for men than women (*p* = 0.04). Age did not correlate to speech perception at any interval (both *p* ≥ 0.2). No correlation was found between the cognitive reserve in total or in any subscore and speech perception at 65 dB at any time (each *p* ≥ 0.2).

### HRQoL

At T1 HRQoL in the subdomain of activity limitations was rated to be the lowest with a mean score of 44.69 (SD 20.28) out of 100 points, contributing to a poor HRQOL in the social domain [mean 45.73 (SD 19.17)]. The highest score was obtained in speech production [mean 65.9 (SD 18.63)]. At T2 improvements were found in the total score from 49.99 (SD 15.87) to 66.22 (SD 14.38) and in all subdomains (all *p* ≤ 0.0001). The highest scores were speech production [78.92 (SD 14.49)] and basic sound perception [69.74 (SD 16.57)], contributing to a high physical domain score [70.95 (SD 13.79)].

Although the total score [mean T2 66.22 (SD 14.38), mean T3 70.7 (SD 16.07); *p* = 0.02] and the scores of the subdomains self-esteem [mean T2 61.06 (SD 15.76), mean T3 67.38 (SD 16.83); *p* = 0.008] and activity limitation [mean T3 61.4 (SD 20.71), mean T3 68.02 (SD 25.28); *p* = 0.04] slightly improved between T2 and T3, this was not significant after Bonferroni correction. In line with that, none of the other subscores significantly improved between T2 and T3 (*p* ≥ 0.06). Comparing HRQoL from T1 to T3, a significant improvement was detected in all subdomains (each *p* < 0.0001).

### Cognitive reserve and depression

The overall CRIq score significantly improved from 111.08 (SD 14.15) to 117.32 (SD 15.04; *p* = 0.01). This indicates a change from medium to high-medium cognitive reserve. This was due to significant improvements in the subcategory leisure activities [mean 117.7 (SD 19.87) at T1; mean 127.66 (SD 27.77) at T3 (*p* = 0.007)]. The subcategories of education (*p* = 0.16) and work (*p* = 0.23) remained stable. Further, the mean level of depressive symptoms did not significantly change over time [2.17 (SD 2.42) at T1 vs 2.4 (SD 2.71) at T3 (*p* = 0.94)].

### Cognitive performance in the total CI group

**Figure 1:**
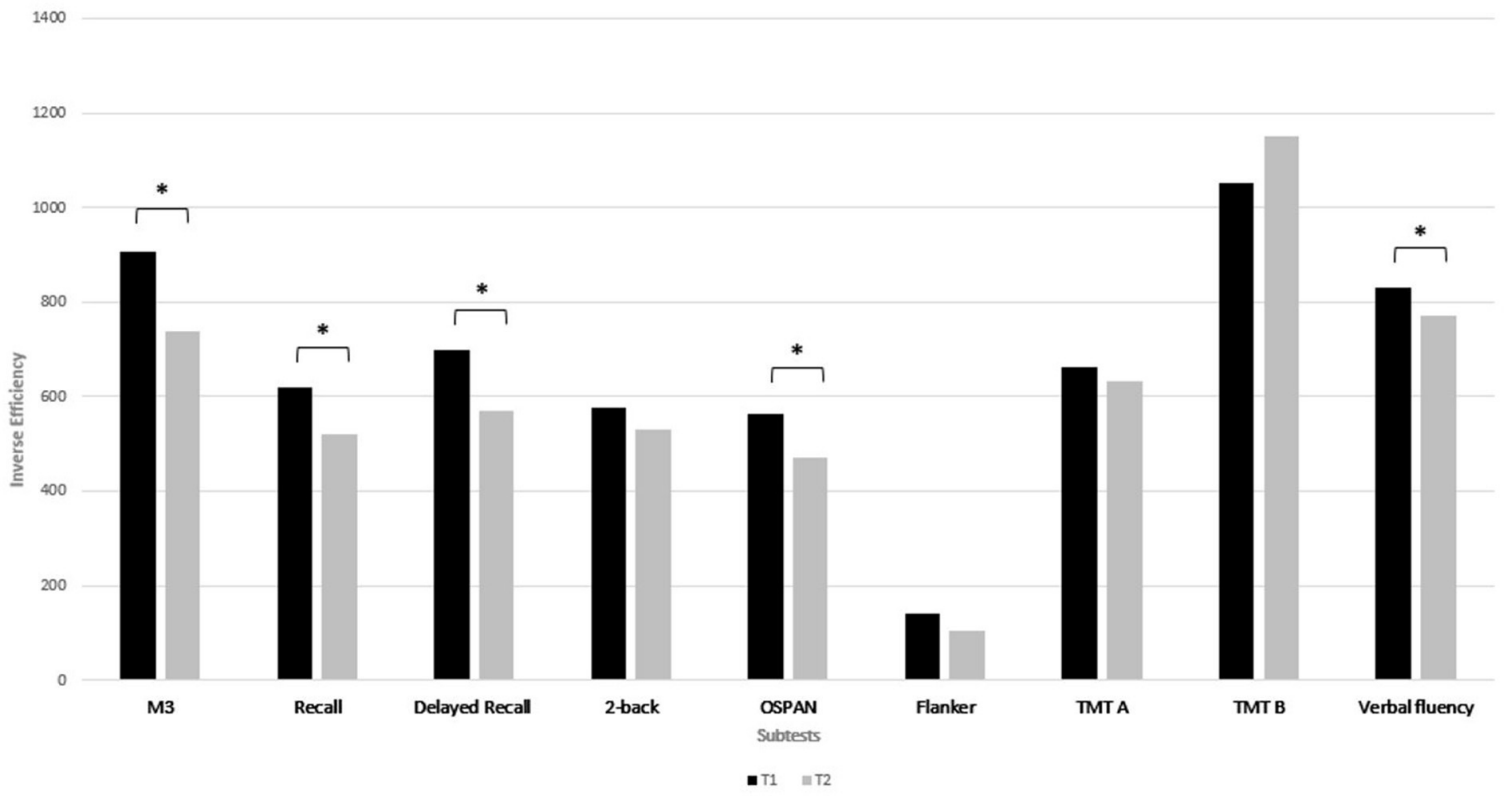
Median of the IE (inverse efficiency) of the neurocognitive subtests at T1 and at T2. A lower IE score indicates a better performance. *Indicates a *p*-value of *p* < 0.005 after Bonferroni correction.

**Figure 2:**
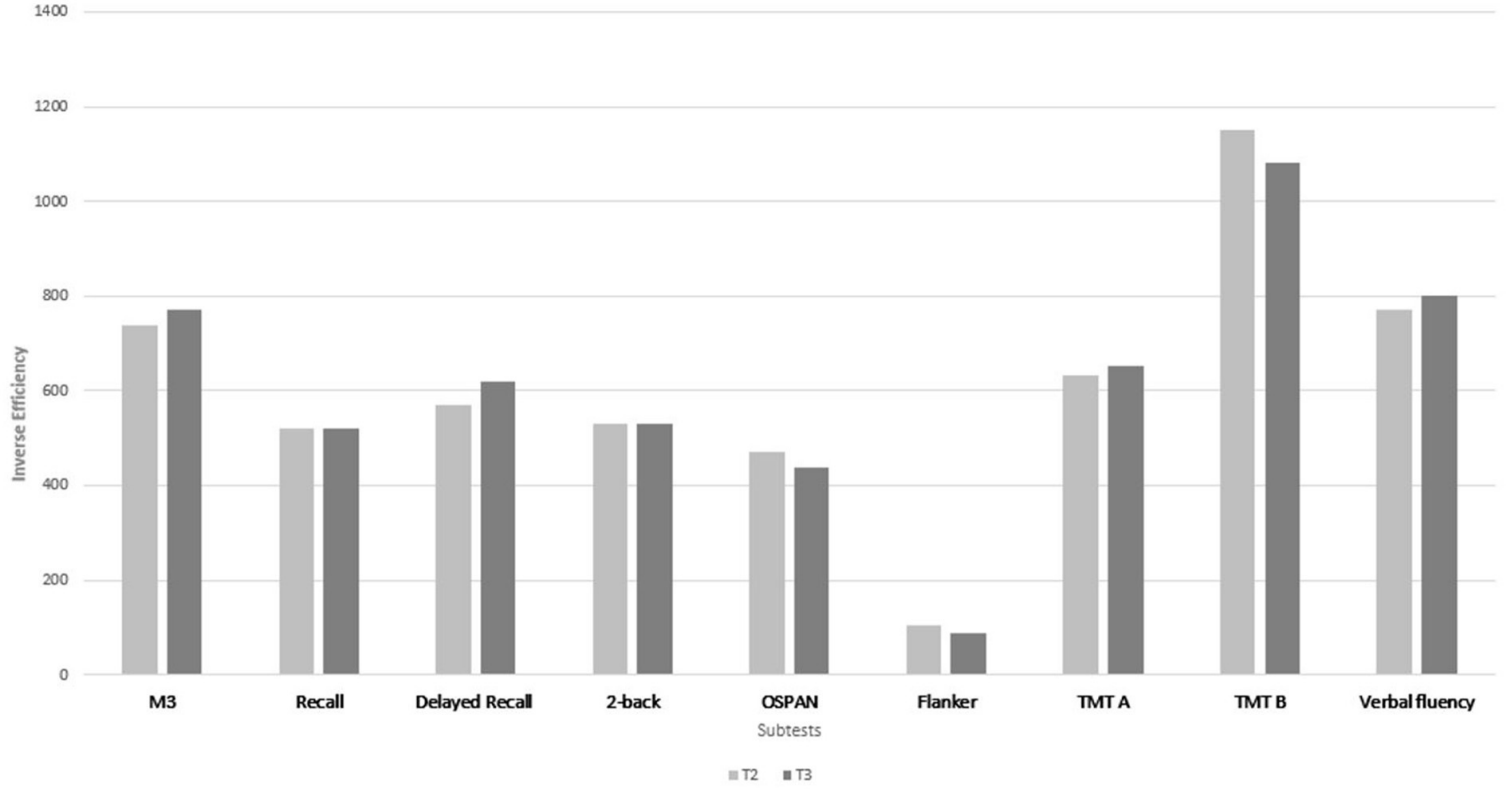
Median of the IE (inverse efficiency) of the neurocognitive subtests at T2 and at T3. A lower IE score indicates a better performance. Between T2 and T3 no significant change was found in any cognitive subtest after Bonferroni correction.

Scores on five of the nine cognitive subtests significantly improved from T1 to T2 (M3, recall, delayed recall, OSPAN and verbal fluency), with a large effect size in the OSPAN task (*d* = 0.8), a medium effect size in the M3 (*d* = 0.69), the delayed recall (*d* = 0.68), and in verbal fluency (*d* = 0.7), and a small effect size in the recall task (*d* = 0.47) (Figure 1). Score on the other four subtests did not change from T1 to T2 (each *p* ≥ 0.04). Between T2 and T3, no further significant benefit was found in any cognitive subtest (each *p* ≥ 0.06) (Figure 2).

**Figure 3:**
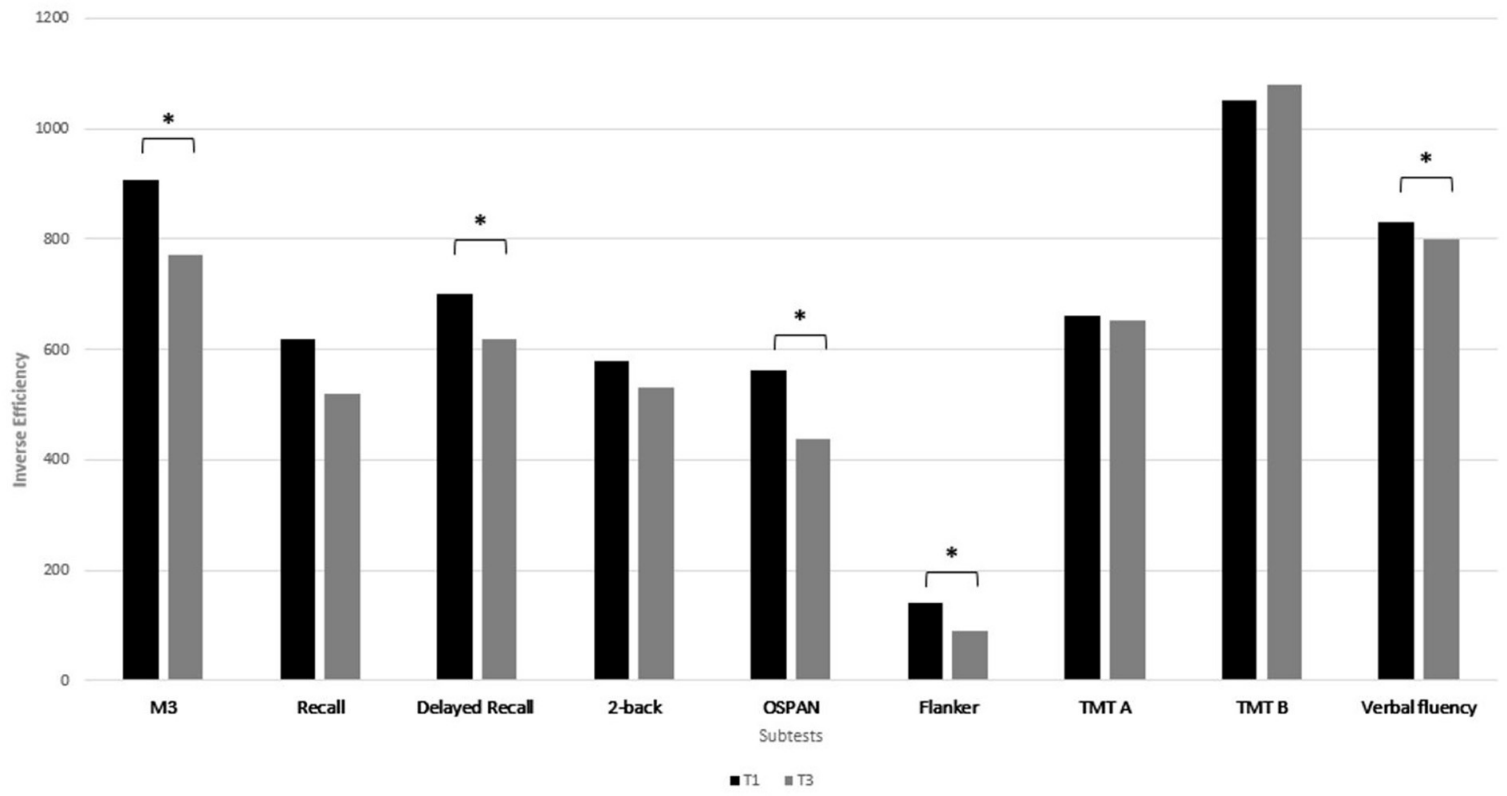
Median of the IE (inverse efficiency) of the neurocognitive subtests at T1 and at T3. A lower IE score indicates a better performance. *Indicates a *p*-value of *p* < 0.005 after Bonferroni correction.

Improvement with a medium effect size from T1 to T3 was seen for attention (*p* = 0.001, *d* = 0.58), delayed recall (*p* = 0.001, *d* = 0.5), for working memory (*p* = 0.001, *d* = 0.54), and inhibition (*p* = 0.002, *d* = 0.5) (Table 2; Figure 3) and with a small effect size for verbal fluency (*p* = 0.004, *d* = 0.43). In contrast, the 2-back only slightly improved (*p* = 0.03), without any significance after multiple correction. Recall (*p* = 0.21), TMT A (*p* = 0.1) and TMT B (*p* = 0.56) were comparable between T1 and T3.

**Table 2:** Median and 68% confidence interval of the Inverse efficiency of the neurocognitive subtests at T1, T2 and at T3.

**Subtest**		**Median**	**68% confidence interval**		**p1**	**p2**	**p3**
M3	T1	906	694.45	1,375.52	0.0001^*^	0.71	0.001^*^
	T2	737.5	603.94	1,033.43			
	T3	771	574.16	1,131.79			
Recall	T1	620	400	700	0.002^*^	0.22	0.21
	T2	520	260	620			
	T3	520	260	700			
Delayed recall	T1	700	533.95	830	0.00004^*^	0.51	0.001^*^
	T2	570	400	821.63			
	T3	620	260	830			
2-back	T1	578	439.77	1,012.07	0.2	0.97	0.03
	T2	532	423.56	839.16			
	T3	530.5	393.1	826.2			
OSPAN	T1	562	359.88	804.71	< 0.0001^*^	0.48	0.001^*^
	T2	472	326.46	664.2			
	T3	439	334.69	781.02			
Flanker	T1	141.5	53.77	236.45	0.04	0.07	0.002^*^
	T2	103.5	38.91	222.12			
	T3	89	47.69	151.08			
TMT A	T1	661	513.61	1,293.91	0.08	0.52	0.1
	T2	632	473	1,190.28			
	T3	652	507	891.92			
TMT B	T1	1,051	738.62	1,897.53	0.68	0.68	0.56
	T2	1,151	701.44	1,857.06			
	T3	1,080.5	778.74	1,937.35			
Verbal fluency	T1	830	735	880	0.00002^*^	0.06	0.004^*^
	T2	770	660	855			
	T3	800	684.62	855			

Comparison between performance at T1 and T2 was labeled with p1, between T2 and T3 with p2 and between T1 and T3 with p3. A lower IE score indicates a better result. A *p*-value < 0.005 indicates significance (

* ) after Bonferroni correction.

The delayed recall and M3 were the tasks, in which most of the subjects improved between T1 and T3 (60.9 % each), followed by the Flanker task (59%). Furthermore, 28 subjects improved in the M3 and 21 subjects in the OSPAN. About 20% of the subjects remained stable in attention, in memory and inhibition and 40% in working memory. Cognitive performance declined only in 15–20% of the subjects in the M3, the Flanker and in the delayed recall and in the OSPAN task in 11%.

### Comparison of cognitive changes in CI recipients and in the general population

**Figure 4:**
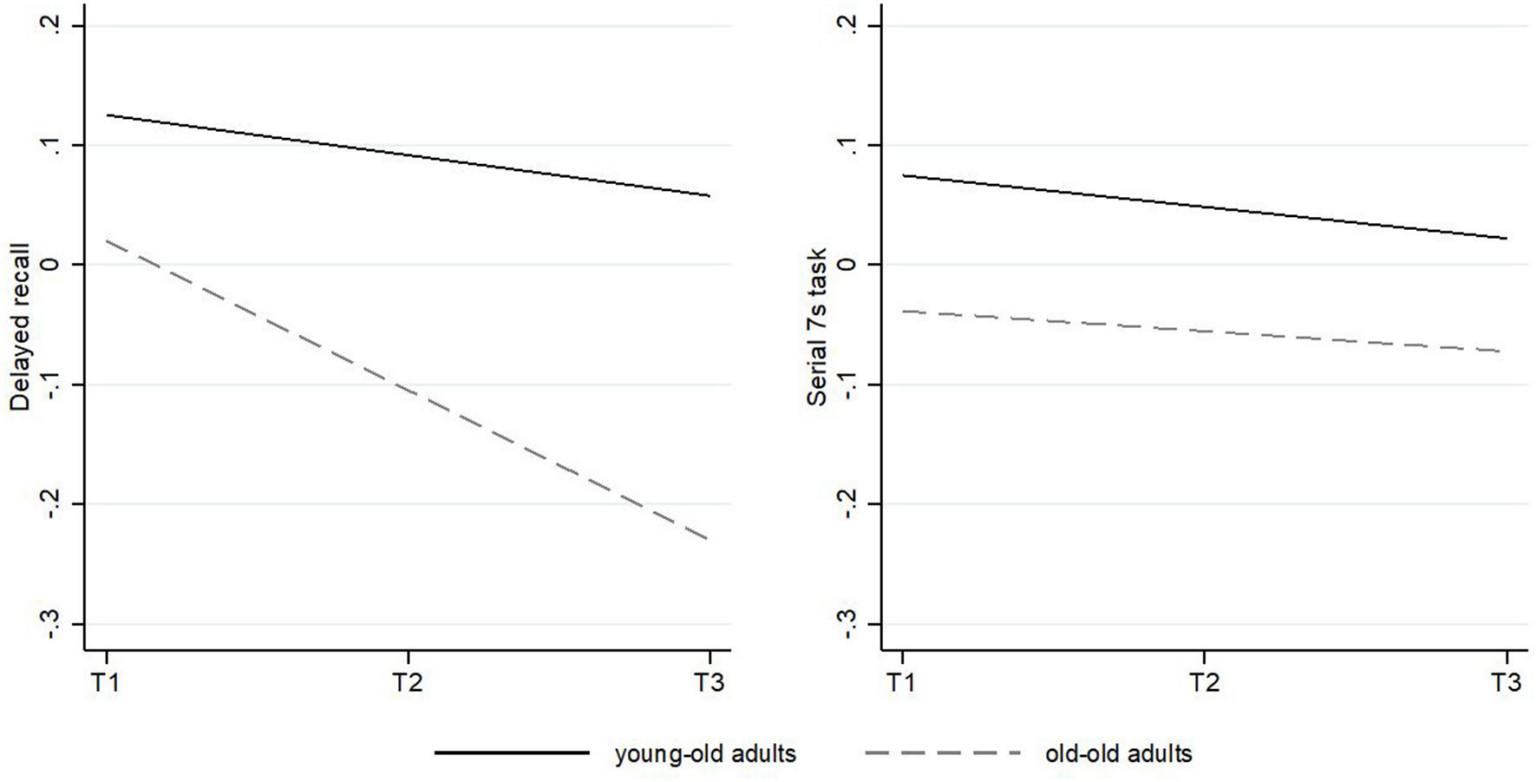
Predicted change of performance in delayed recall and the Serial 7s task in the SHARE sample over time. The solid slope shows the trajectory for young-old adults with an average age mean of 60.51 years; the dashed slope shows the trajectory for old-old adults with an average age mean of 69.59 years. Slopes are controlled for all covariates.

We report our findings based on two models. The first model (Model 1) predicted variation in cognitive measures as a function of time and of the other covariates. The second model (Model 2) included an additional time X age interaction to explore effects of age on change over time. With regard to **memory**, Model 1 (main effect) intercepts indicated that average participants in SHARE started from a higher average delayed recall level than the CI recipients. It also indicated that delayed recall was lower for each year of increased age (−0.022) and for males (−0.270) and higher for better educated individuals (0.143) in the SHARE group. The time slope showed a linear decrease in delayed recall for each measurement (−0.076), indicating an overall decline in this cognitive domain over the observational period. Model 2 (interaction affect) revealed a significant time × age interaction (−0.010) suggesting that declines in delayed recall over time were stronger with higher age. Model 1 in the CI sample provided a negative effect of age (−0.034) on delayed recall. A different pattern emerged with respect to the time slope, which showed an increase (0.169) in delayed recall over the observational period. Model 2 did not reveal a significant time × age interaction, suggesting that positive changes were not dependent on chronological age (see Table 3A; Figure 4).

**Table 3A:** Multilevel regression growth models predicting change in delayed recall in the CI recipients and the SHARE sample.

	**Cochlear implant recipients**				**SHARE sample**			
	**Model 1**		**Model 2**		**Model 1**		**Model 2**	
	**B**	**SE**	**B**	**SE**	**B**	**SE**	**B**	**SE**
Intercept	0.011	0.168	0.011	0.168	0.203^***^	0.038	0.203^***^	0.038
Age	−0.034^*^	0.014	−0.030	0.016	−0.022^***^	0.004	−0.012^*^	0.004
Male	−0.466	0.261	−0.496	0.261	−0.270^***^	0.050	−0.270^***^	0.050
Education	0.168	0.163	0.401	0.249	0.143^***^	0.042	0.143^***^	0.042
Time	0.169^*^	0.068	0.169^*^	0.068	−0.076^***^	0.016	−0.076^***^	0.015
Time × age			−0.005	0.008			−0.010^***^	0.002

B, unstandardized regression coefficient; SE, standard error. Intercept reflects the outcomes when all predictors are equal to zero (i.e., average age, female, average education, first measurement).

* *p* < 0.05;

*** *p* < 0.001.

**Figure 5:**
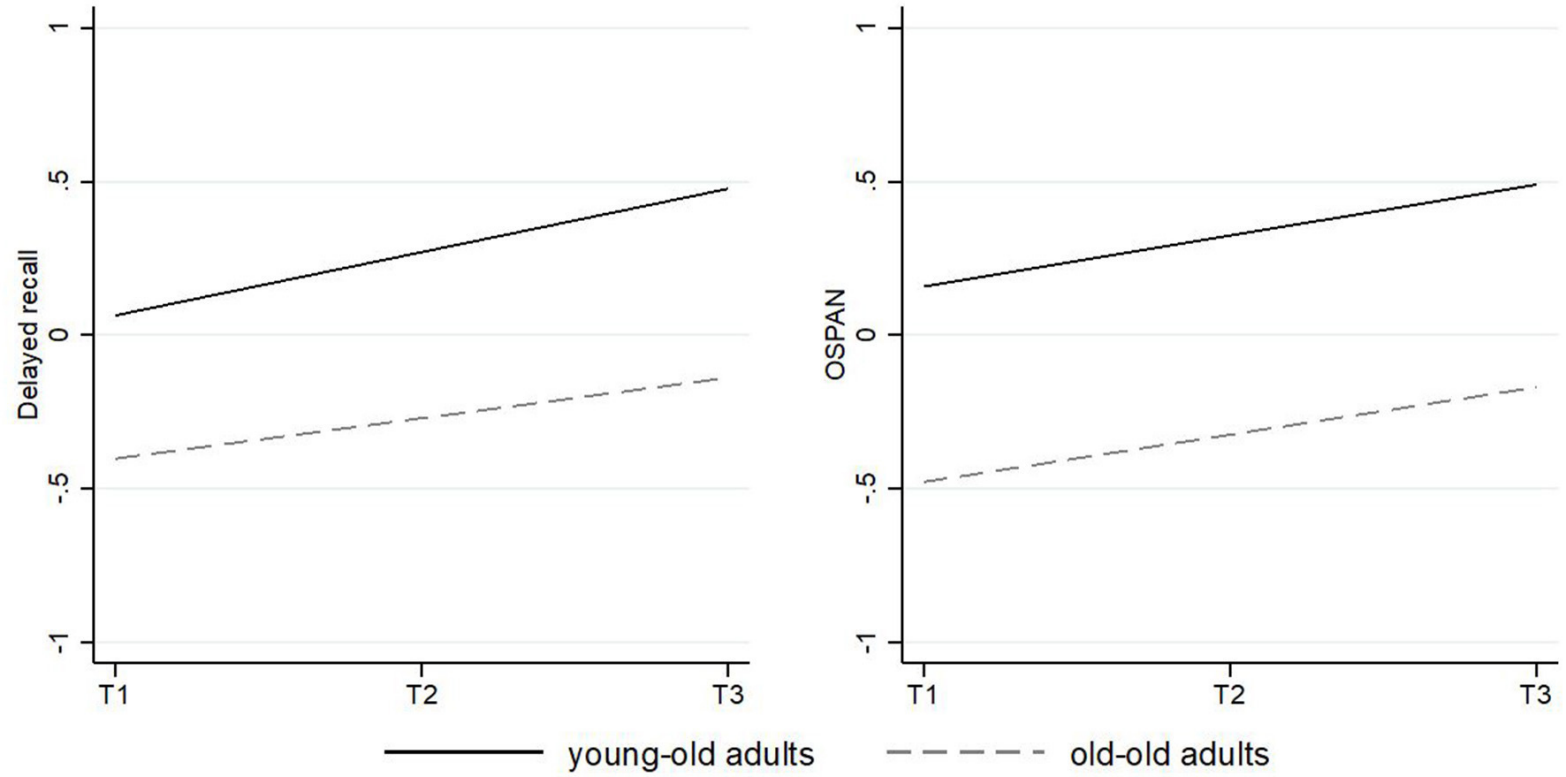
Predicted change of performance in delayed recall and the OSPAN in the cochlear implant sample over time. The solid slope shows the trajectory for young-old adults with an average age mean of 56.73 years; the dashed slope shows the trajectory for old-old adults with an average age mean of 72.50 years. Slopes are controlled for all covariates.

Concerning **working memory**, intercepts in model 1 (main effect) indicated that the average SHARE participants had higher initial levels in working memory than the CI recipients. The serial 7s task score was lower for older adults (−0.011), whereas positive associations were found for males (0.104) and participants with a better education (0.113). The time slope did not show a significant decrease in the serial 7s task, indicating overall stability in the SHARE data in this domain over time. Model 2 did not provide a significant time × age interaction, indicating that longitudinal changes in the serial 7s task did not differ for older adults. Regarding the OSPAN measure in the CI sample, model 1 indicated a negative effect of age (−0.040) and a positive effect of higher education (0.559). Again, a different pattern emerged with respect to change over time suggesting positive changes in OSPAN scores over time (0.167). We did not find a time × age interaction (see Table 3B; Figure 5).

**Table 3B:** Multilevel regression growth models predicting change in the OSPAN (cochlear implant recipients) and in the serial 7s (SHARE sample).

	**Cochlear implant recipients**				**SHARE sample**			
	**Model 1**		**Model 2**		**Model 1**		**Model 2**	
	**B**	**SE**	**B**	**SE**	**B**	**SE**	**B**	**SE**
Intercept	−0.147	0.162	−0.147	0.162	−0.027	0.038	−0.027	0.038
Age	−0.040^**^	0.014	−0.038^*^	0.015	−0.011^**^	0.004	−0.012^*^	0.005
Male	−0.057	0.263	−0.057	0.263	0.104^*^	0.050	0.104^*^	0.050
Education	0.559^**^	0.165	0.559^**^	0.164	0.113^**^	0.041	0.113^**^	0.041
Time	0.167*^**^	0.046	0.167*^**^	0.046	−0.022	0.017	−0.022	0.017
Time × age			−0.002	0.005			−0.001	0.002

B, unstandardized regression coefficient; SE, standard error. Intercept reflects the outcomes when all predictors are equal to zero (i.e., average age, female, average education, first measurement).

* *p* < 0.05;

** *p* < 0.01; ***p < 0.001.

After accounting for subjective assessment of hearing impairment, all reported findings remained robust except for the time slope showing a negative significant decrease in the serial 7s task (see Supplementary Table S1).

The **performance of individual subjects** in the four most important neurocognitive subtests (Flanker, M3, OSPAN, and delayed recall) was analyzed across intervals. Due to the high variability in the performance among the individuals, only CI recipients' data that either increased, decrease, or remained stable in at least three out of the four tests were reported.

Performance on three or four tests improved in 21 subjects, remained stable in four subjects, and declined in only one subject between T1 and T2; improved in five subjects, remained stable in four subjects, and declined in three participants between T2 and T3; and improved in 19 subjects and declined in only one subject between T1 and T3.

**Figure 6:**
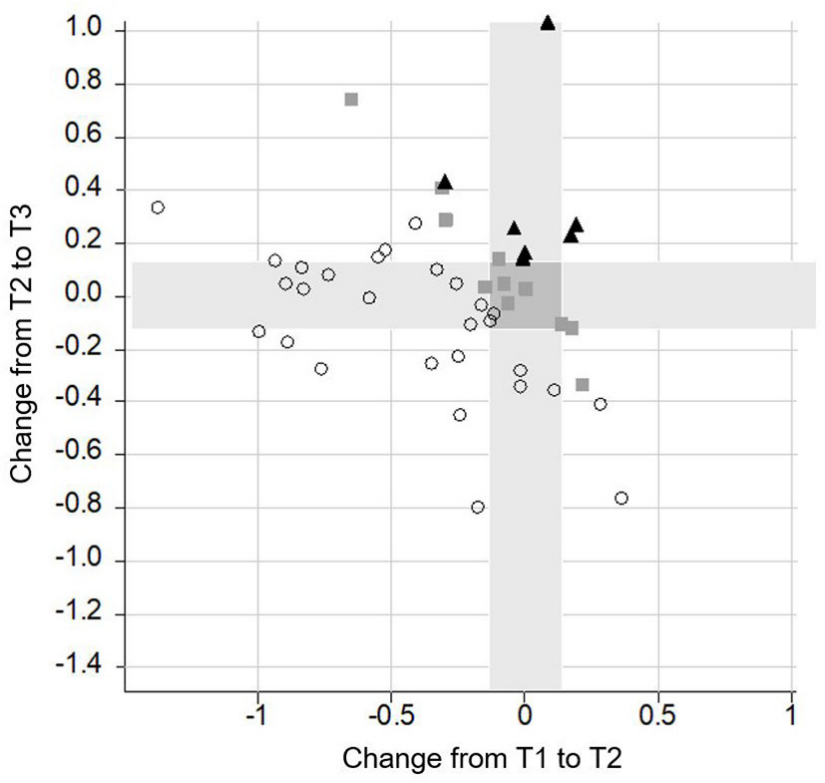
Change of performance in the **M3** task. Lower scores indicate a better performance. Each symbol represents a person according to their change from T1 to T2 (*x*-axis) and T2 to T3 (*y*-axis). The overall change of the person from T1 to T3 is indicated by different shapes (▴ = poor performance; ■ = stable performance; ○ = improved performance). All symbols right from the vertical grey bar indicate a decrease in performance from T1 to T2. All symbols above the horizontal grey bar represent a poorer performance from T2 to T3. Giving an example, the lowest dot on the right side indicates a decrease in performance from T1 to T2. In contrast, from T2 to T3 there was an increase in performance. In total, the subject improved from T1 to T3 and therefore, it was labeled by a dot. Furthermore, the highest square which you can find is on the left side of the vertical grey bar. This means that it increased from T1 to T2. From T2 to T3 performance decreased, as the square is above the horizontal grey bar. In total, this subject remained stable and therefore, it was labeled by a square.

**Figure 7:**
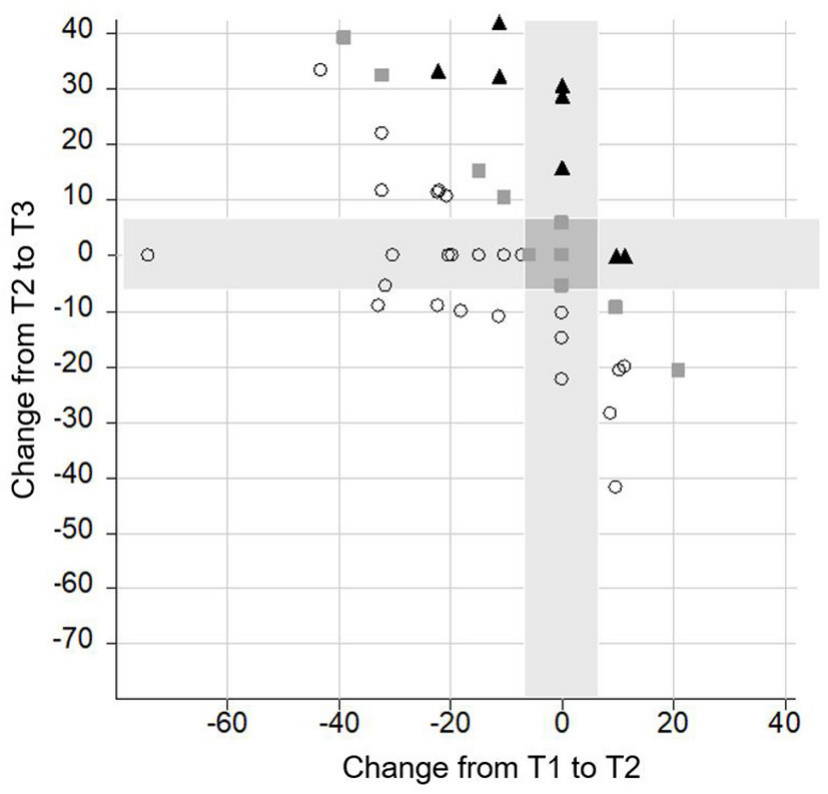
Change of performance in the **delayed recall** task. Lower scores indicate a better performance. Each symbol represents a person according to their change from T1 to T2 (*x*-axis) and T2 to T3 (*y*-axis). The overall change of the person from T1 to T3 is indicated by different shapes (▴ = poor performance; ■ = stable performance; ○ = improved performance).

**Figure 8:**
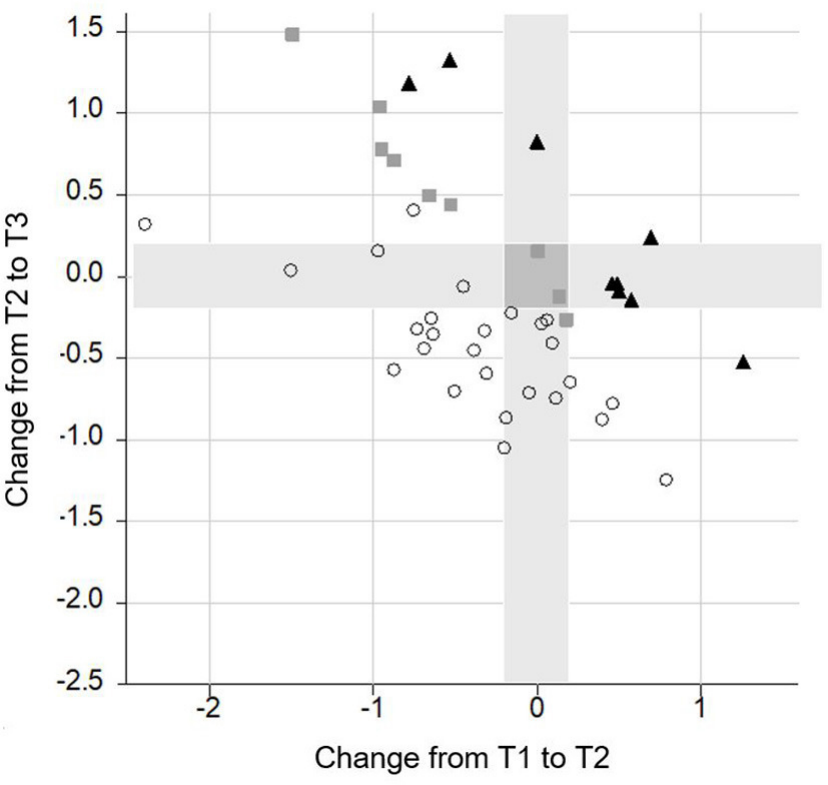
Change of performance in the **Flanker** task. Lower scores indicate a better performance. Each symbol represents a person according to their change from T1 to T2 (*x*-axis) and T2 to T3 (*y*-axis). The overall change of the person from T1 to 3 is indicated by different shapes (▴ = poor performance; ■ = stable performance; ○ = improved performance).

**Figure 9:**
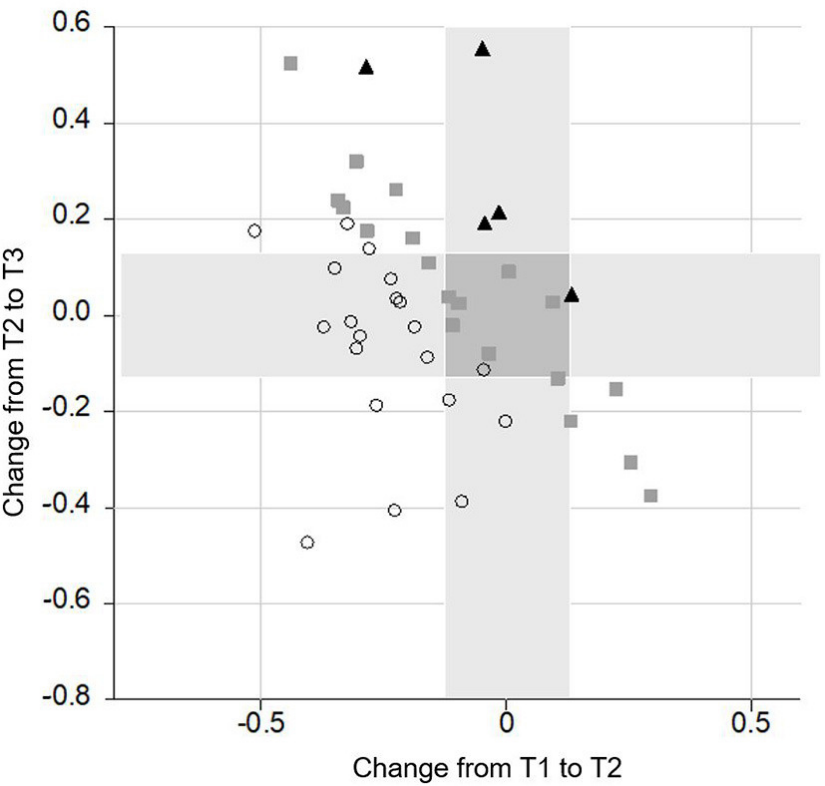
Change of performance in the **OSPAN** task. Lower scores indicate a better performance. Each symbol represents a person according to their change from T1 to T2 (*x*-axis) and T2 to T3 (*y*-axis). The overall change of the person from T1 to 3 is indicated by different shapes (▴ = poor performance; ■ = stable performance; ○ = improved performance).

Data analysis further revealed that only a minority of the CI recipients had a poorer performance in one (*n* = 17) or two (*n* = 6) subtests between T1 and T3. With regard to the different subtests, some CI recipients had a gain between T1 and T2 and a poorer performance between T2 and T3. This was the case in nine subjects in the M3, in 10 CI recipients in the Flanker and in 11 subjects in the OSPAN and in the delayed recall. Notably, this decline did not outweigh the gain in performance achieved in the long-term follow-up, so that at T3 the majority of the CI recipients scored equally or even better than preoperatively (see Figures 6–9).

Subjects with a poorer T1 performance also had worse results at T2 and T3 in all subtests (each *p* ≤ 0.0001) although improvement was significantly greater in these subjects. This was the case at T2 for the M3 (tau = −0.39, *p* < 0.0001), the 2-back (tau = −0.3, *p* = 0.003), the OSPAN (tau = −0.52, *p* < 0.0001), the Flanker (tau = −0.38, *p* = 0.0001), and the TMT A (tau = −0.39, *p* = 0.0001); and at T3 for the M3 (tau = −0.3, *p* = 0.002), the OSPAN (tau = −0.32, *p* = 0.002), the Flanker (tau = −0.55, *p* < 0.001) and the TMT A (tau = −0.55, *p* < 0.001).

At T3, after adjusting for age and education (each *p* ≤ 0.005), the baseline score was the most important predictive in all cognitive subtests. Preoperative and postoperative speech perception score in quiet at 65 dB did not correlate with any cognitive subtest (each *p* ≥ 0.13 and each *p* ≥ 0.19). This was also true for the improvement of cognitive functions at T1 and T3 (each *p* ≥ 0.18).

### Covariates

**Age** had an impact on cognition pre- and post-implantation. This was the case for the TMT A (*p* ≤ 0.001), the TMT B (*p* ≤ 0.002), and the Flanker tasks (*p* ≤ 0.001) at T1 and T3 as well as at T2 in the TMT A (*p* = 0.001). Improvement in cognitive functions did not correlate with age in any subtest (each *p* ≥ 0.06). Men and women performed equally in all cognitive subtests (each *p* ≥ 0.05) except on the 2-back task after 12 months, where men outperformed women (*p* = 0.00006). However, improvement in the 2-back performance was greater for women than for men between T2 and T3 (*p* = 0.002). For all other cognitive subtasks, the improvement was comparable between men and women (each *p* ≥ 0.06). Mean **educational level** was 11.96 (SD 2.09) ranging from 8 to 17 years. Interaction of age, sex, and educational background was detected for the 2-back and the verbal fluency task at T3. Whereas in the 2-back task educational background was more important for men than for women (2-back *p* = 0.02), in the verbal fluency task education had only an impact on performance in women (*p* = 0.03).

## Discussion

Cognitive decline in age takes many years and there is a high variability in cognitive trajectories in the general population (69). Thus, the effects of auditory rehabilitation on cognition are difficult to assess. Only a few studies analyzed CI users' long-term cognitive performance with a focus on the single subject and in the light of a suitable control group.

In the present study, CI recipients had a significantly better cognitive performance at T3 than at T1. This was most evident in delayed recall, attention, and working memory assessed by the OSPAN task; but also in verbal fluency and inhibition. Performance on other cognitive subdomains such as the 2-back also improved but were no longer significant after Bonferroni correction. In contrast, performance on the TMT A, the TMT B, and the recall task remained without change.

Improvements in attention and in the total RBANS-H score were also described by Mertens et al., whose participants were 24 CI recipients (mean age of 72 years) when assessed 14 months after cochlear implantation (31).

Cosetti et al., who reported on a long-term follow-up of 3.7 years after implantation, found an enhanced cognitive performance in 70% of the 20 cognitive tasks of which some were taken from the Wechsler Adult Intelligent Scale and from the RBANS (28). In contrast, Sarant et al. did not see any change in cognitive test scores in the Cogstate battery in 59 CI recipients (mean age of 72.3 years) after 18 months of CI use. Only the subgroup of men with lower educational achievement significantly improved in executive functions in the Groton Maze learning test (20).

In order to analyze cognitive changes in the follow-up, multiple assessments might be helpful to draw a slope (70). Multiple cognitive assessments in the follow-up after cochlear implantation have rarely been studied. In the present study, mean cognitive performance showed a significant enhancement after 12 months and remained stable at up to 5 years. Our data support those of Ohta et al., who also found a peak 12 months after implantation (in 21 CI recipients aged between 65–80 years 12 months after cochlear implantation) and a plateau which remained stable at up to 24 months. Unfortunately, no analysis was done on the different subtests of the MMSE to see which cognitive subdomain benefits the most (35). In contrast to earlier results by Mosnier et al. (23), in their latest data set no significant improvement was found after 12 months of CI use. Scores even declined in the long-term follow-up in the clock drawing test, the d2 test, the TMT tasks and in the MMSE, while scores on the 5 word-test and categorial verbal fluency remained stable (32).

Further, changes in cognitive function after auditory rehabilitation have mostly been discussed in light of whole samples rather than for individuals themselves. It is important to note that change in terms of mean-level change only refers to average increase or decrease within a specific group over time. However, lack of mean-level change does not rule out the possibility that substantial individual-level change exists. For example, individuals may increase and decrease offsetting each other's change. Given that individual variability in cognitive function is greater in the older population (71, 72), and even greater in clinical populations with chronic diseases, this needs to be considered (43, 73). So far, only two studies have analyzed subjects' individual trends. Cosetti et al. described the individual performance of each single CI recipient (*n* = 7) in any of the tests applied, by either a positive or a negative change. One subject improved in five of 15 subtests, two subjects in six or nine of 17 subtests and four subjects in seven up to 10 of 20 subtests (28). Mosnier et al. clustered their sample into MCI subjects, cognitively healthy individuals and subjects suffering from dementia. Of the 29 MCI participants 19 remained stable, 10 returned to normal cognition, and only one developed dementia at a mean follow-up of 6.8 years of CI use. At the same follow-up time amongst participants with normal preoperative cognition 26 remained stable and 32% developed mild cognitive impairment. Interestingly, the proportion of subjects with preoperative mild cognitive impairment included in Mosnier et al. was 45%, which is relatively high compared to the estimated 12–15% in the general population of people aged ≥60 years and might be country- or region-specific (74, 75). In contrast, in mean cognitive test scores did not improve at 12-months post-CI and a decline in the Mini Mental Status Examination, the Clock Drawing Test, the D2 and the Trail Making Test A and B was observed in the follow-up.

Results of the present study indicate that cognitive function underlies individual variability between the test intervals and according to the different subtests. In general, the majority of the subjects showed an enhancement in overall performance, only a few a total decrease; however, some subjects increased in the first interval and decreased or remained stable later or even reversed. Subjects with a worse preoperative neurocognitive performance enhanced the most.

Other authors also claim that individuals with a poor baseline performance show the greatest improvement (18, 23, 26). Therefore, one may speculate that a CI should not be denied to people with mild cognitive impairment. Recent studies have explored the effect of hearing device use on the cognitive function of people with cognitive impairment (76–78), nonetheless more data on this is needed.

### Long-term effects of cochlear implantation and comparison

To better judge the cognitive changes in the CI group and considering the high cognitive variability in age, we included a huge control group and compared two cognitive tests, one for memory and one for working memory with similar measures from a large representative data set of the Survey of Health, Ageing and Retirement in Europe (SHARE). We also estimated multilevel growth models to explore the average cognitive change in both samples. Three key findings emerged from these analyses.

Firstly, when only focusing on the CI users, positive changes in delayed recall and working memory remained robust within a rigorous and well-controlled longitudinal design. This indicates that positive cognitive changes occurred over longer observational periods even when controlling for sociodemographic characteristics. This adds to existing research in the field that has primarily explored changes after cochlear implantation within pre-post study designs.

Secondly, positive time slopes in cognition among CI users were not dependent on chronological age. This rules out the possible explanation, that only middle-aged adults would benefit in terms of cognition. This finding is remarkable given that biomechanical cognitive abilities typically show age-related declines into later life (61). However, a robust body of knowledge has proven that cognitive plasticity occurs even until very late in life (48) and our findings suggest that cochlear implantation may play a potential role in contributing to such plasticity.

Thirdly, the importance of our findings from CI users is further underscored after comparison of this specific study population with the SHARE data. In these secondary data analyses, we found the expected age-related negative trajectories over time in memory (delayed recall task) and some degree of stability in working memory performance (Serial 7s task). In addition, the SHARE respondents showed an even steeper decline in memory with increasing age. Treating the secondary data as an approximation of a nonexperimental comparison group, we argue that these findings demonstrate the beneficial effects of CI use among older adults. This approach, however, is limited due to different cognitive base-levels and nonequivalent dependent measures between the CI and the SHARE group. Another issue pertains to the lack of objective audiometric assessments in the SHARE data to better control for the potential influence of hearing impairment on cognition over time. Supplementary analyses indicated that the overall findings were robust when including the subjective assessment of hearing which is included in the SHARE data. However, hearing loss is often underestimated in hearing-impaired especially in older subjects (79, 80). Audiometric assessments are clearly needed in future research with secondary data. Moreover, future studies would benefit from constructing propensity scores that balance treatment and control groups on potentially relevant baseline variables.

Considering that hearing loss is associated with a faster cognitive decline (16, 81), the observation that cognition improves after implantation and that such improvement is maintained at 12 months of CI use is promising. We should encourage older people to treat age-related hearing loss (82). However, the findings of the present study have to be critically discussed.

First of all, one has to keep in mind that not all subjects who were included preoperatively could be followed-up. Thus, one might argue that only subjects with an active lifestyle and better cognitive functions agreed to do the re-evaluation of the cognitive performance after 60 months. Thus, subjects who did not improve in the same way might be underrepresented although there was no statistical difference between the 50 subjects included and the total CI group. Further, this bias of nonparticipation might be the case in any study protocol.

Further, all CI users in the present study received an intensive auditory rehabilitation schedule as defined by the guidelines of the German society of Ear, Nose and Throat Medicine (83). So, the interactive effects of the behavioral speech and language therapy on cognitive aging cannot be ruled out. This has to be stressed as cognitive enhancement in the present study as well as in the literature is mainly reported during the first year after implantation, when rehabilitation usually takes place. In addition, the level of leisure activities as shown in the CRIq significantly increased. One may argue that better audibility motivates the CI recipient to take part in leisure activities more frequently. This, in turn might contribute to better cognitive abilities independently of education and occupation as shown in the Cambridge Center for Ageing and Neuroscience study (84).

Therefore, cochlear implantation might have a booster effect on cognition which might decline in the follow-up. Data by our group as well as in the literature did not show a correlation between speech perception and cognition or between the improvement in speech perception and in cognitive performance (32, 44, 85). Thus, it is not clear whether this enhancement is really direct due to an improvement in auditory abilities or whether it is indirect due to a general stimulating effect. Speech recognition alone might not be sufficient and social interaction might be crucial to enhance cognition (35, 44). Further, rehabilitative training might have also triggered the better cognitive performance after 1 year.

In addition, even if the performance in the total CI group significantly improved, cognitive changes varied greatly between the single subjects. Thereby, the number of subjects included—although being one of the largest in this field—might be too small due to the high inter-individual variability of cognitive aging. Studies with larger sample sizes need to be performed to control for the various participants' characteristics and to minimize the impact of these features on outcome measures.

What's more, we do not know to what extent laboratory-based cognitive tasks can predict real-life outcomes in older adults: older adults often function competently in complex everyday situations despite age-related deficits on laboratory-based cognitive tasks (86). Several factors have been identified as influencing everyday activities realization, including physical and cognitive functioning (87). However, there is little evidence that interventions improve performance on distantly related tasks or that training improves everyday functioning in later life (88). A classic study from Ball et al. (89) assessed the effects of cognitive training interventions on older adults and found that cognitive training did not affect daily functioning over 2 years. In their follow-up study, they explored 10-year effects of cognitive training on cognition and everyday functioning in older adults (90). Findings suggest slower declines in performing IADLs (Instrumental Activities in Daily Living) in intervention groups over 10-years; however, effects were modest and even absent with respect to performance-based everyday functioning tests.

Lastly, although the present study's follow-up time is longer than in most similar studies, it still might be too short to determine if a CI can arrest or even reverse cognitive decline. Dementia takes multiple years to develop, and cognitive decline might only be observed in studies which have a follow-up time of up to, or even longer than, 10 years.

## Conclusion

Auditory rehabilitation by cochlear implantation seems to stimulate the plasticity of the brain within the first year after implantation leading to an improvement in some cognitive functions in the follow-up in the total group in comparison with data of a representative sample. However, large multicenter studies on CI recipients with a long-term follow-up of up to 10 years or even more must be undertaken to confirm the present data. To allow comparability, the development of a standard diagnostic protocol including cognitive assessment tools adapted to severe hearing-impaired will be the first step.

## Data availability statement

The original contributions presented in the study are included in the article/Supplementary material, further inquiries can be directed to the corresponding author.

## Ethics statement

The studies involving human participants were reviewed and approved by Ruhr-University Bochum, Germany. The patients/participants provided their written informed consent to participate in this study.

## Author contributions

CV and JT designed the study. LG selected the subjects and collected a part of the data. CV, IH, SK, and LG analyzed and evaluated the data. CV wrote the manuscript with contributions from LG and critical feedback from SD and JT. All authors contributed to the article and approved the submitted version.

## Funding

This paper uses data from SHARE Waves 6, 7, and 8 10.6103/SHARE.w7.800, 10.6103/SHARE.w8.800, 10.6103/SHARE.w8ca.800, see Börsch-Supan et al. (62) for methodological details. The SHARE data collection has been funded by the European Commission, DG RTD through FP5 (QLK6-CT-2001-00360), FP6 (SHARE-I3: RII-CT-2006-062193, COMPARE: CIT5-CT-2005-028857, SHARELIFE: CIT4-CT-2006-028812), FP7 (SHARE-PREP: GA N°211909, SHARE-LEAP: GA N°227822, SHARE M4: GA N°261982, DASISH: GA N°283646) and Horizon 2020 (SHARE-DEV3: GA N°676536, SHARE-COHESION: GA N°870628, SERISS: GA N°654221, SSHOC: GA N°823782, SHARE-COVID19: GA N°101015924) and by DG Employment, Social Affairs & Inclusion through VS 2015/0195, VS 2016/0135, VS 2018/0285, VS 2019/0332, and VS 2020/0313. Additional funding from the German Ministry of Education and Research, the Max Planck Society for the Advancement of Science, the U.S. National Institute on Aging (U01_AG09740-13S2, P01_AG005842, P01_AG08291, P30_AG12815, R21_AG025169, Y1-AG-4553-01, IAG_BSR06-11, OGHA_04-064, HHSN271201300071C, RAG052527A) and from various national funding sources is gratefully acknowledged (see www.share-project.org).

## Conflict of interest

Authors CV, JT, and SD have received reimbursement of scientific meeting participation fees and accommodation expenses, as well as honoraria for preparing continuing medical education events and funding for research projects that they initiated, from MED-EL. The remaining authors declare that the research was conducted in the absence of any commercial or financial relationships that could be construed as a potential conflict of interest.

## Publisher's note

All claims expressed in this article are solely those of the authors and do not necessarily represent those of their affiliated organizations, or those of the publisher, the editors and the reviewers. Any product that may be evaluated in this article, or claim that may be made by its manufacturer, is not guaranteed or endorsed by the publisher.
