# Supplementary material for: Can cochlear implantation prevent cognitive decline in the long-term follow-up?
Source: Front Neurol. 2022 Oct 20;13:1009087. doi: 10.3389/fneur.2022.1009087 (PMC9631779; doi:10.3389/fneur.2022.1009087)
Supplement: Supplementary file 1 [file Table_1.docx]

|  | **Delayed recall** | | | |  | **Serial 7s** | | | |
| --- | --- | --- | --- | --- | --- | --- | --- | --- | --- |
|  | Model 1 | | Model 2 | |  | Model 1 | | Model 2 | |
|  | *B* | *SE* | *B* | *SE* |  | *B* | *SE* | *B* | *SE* |
| *Intercept* | .208*** | .037 | .208*** | .037 |  | .014 | .038 | .014 | .038 |
| *Age* | -.022*** | .004 | -.015** | .005 |  | .002 | .004 | .001 | .005 |
| *Male* | -.261*** | .050 | -.261*** | .051 |  | .080 | .051 | .080 | .051 |
| *Education* | .211*** | .042 | .113** | .041 |  | .154*** | .042 | .155*** | .042 |
| *Subjective hearing* | -.050 | .027 | -.50 | .027 |  | -.02 | .027 | -.02 | .027 |
| *Time* | -.090*** | .016 | -.090*** | .015 |  | -.050** | .017 | .050** | .017 |
| *Time × Age* |  |  | -.008** | .003 |  |  |  | .001 | .003 |

**Table S1** Multilevel Regression Growth Models Predicting Change in delayed recall and the Serial 7s task in the SHARE sample with subjective evaluation of hearing (1 = excellent to 5 = poor) as additional covariate *Note*. B = unstandardized regression coefficient; SE = standard error. Intercept reflects the outcomes when all predictors are equal to zero (i.e., average age, female, average education, average hearing, first measurement). ^*^*p* <.05; ^**^*p* <.01; ^***^*p* <.001.
